# A phenomics approach to the analysis of the influence of glutathione on leaf area and abiotic stress tolerance in Arabidopsis thaliana

**DOI:** 10.3389/fpls.2013.00416

**Published:** 2013-11-05

**Authors:** Daniel Schnaubelt, Philipp Schulz, Matthew A. Hannah, Rosita E. Yocgo, Christine H. Foyer

**Affiliations:** ^1^Centre of Plant Sciences, Faculty of Biology, University of LeedsLeeds, UK; ^2^Bayer CropScience NVGent, Belgium; ^3^Botany Department, Forestry and Agricultural Biotechnology Institute, University of PretoriaPretoria, South Africa

**Keywords:** abiotic stress tolerance, glutathione synthesis, root architecture, lateral root density, leaf area

## Abstract

Reduced glutathione (GSH) is an abundant low molecular weight plant thiol. It fulfills multiple functions in plant biology, many of which remain poorly characterized. A phenomics approach was therefore used to investigate the effects of glutathione homeostasis on growth and stress tolerance in *Arabidopsis thaliana*. Rosette leaf area was compared in mutants that are either defective in GSH synthesis (*cad2*, *pad2*, and *rax1*) or the export of γ-glutamylcysteine and GSH from the chloroplast (*clt*) and in wild-type plants under standard growth conditions and following exposure to a range of abiotic stress treatments, including oxidative stress, water stress, and high salt. In the absence of stress, the GSH synthesis mutants had a significantly lower leaf area than the wild type. Conversely, the* clt *mutant has a greater leaf area and a significantly reduced lateral root density than the wild type. These findings demonstrate that cellular glutathione homeostasis exerts an influence on root architecture and on rosette area. An impaired capacity to synthesize GSH or a specific depletion of the cytosolic GSH pool did not adversely affect leaf area in plants exposed to short-term abiotic stress. However, the negative effects of long-term exposure to oxidative stress and high salt on leaf area were less marked in the GSH synthesis mutants than the wild type. These findings demonstrate the importance of cellular glutathione homeostasis in the regulation of plant growth under optimal and stress conditions.

## INTRODUCTION

Environmental stresses severely limit plant growth and decrease the predictability of crop yields for the farmer. Abiotic stress often has a greater impact on crop productivity than genotypic effects. Enhancing stress tolerance is therefore a major second-generation trait target for crop improvement programs. Plant stress responses are complex traits regulated by large numbers of genes and quantitative trait loci (QTL). This complexity has restricted the success of conventional breeding approaches. Similarly, transgenic approaches to enhancing tolerance to complex stresses such as drought have not as yet significantly reduced environmentally related yield losses under field conditions (Lawlor, 2013). A greater understanding of the mechanisms that restrict the growth of plants in response to the imposition of abiotic stress is required to facilitate development and molecular breeding of crop varieties with enhanced stress tolerance traits.

Enhanced cellular oxidation is a common feature of the plant response to stress. Oxidative signaling underpins plant responses to stress and is intimately associated with hormone signaling pathways that regulate plant growth, senescence, and cell death responses. The thiol tripeptide, glutathione (GSH; γ-glutamyl-L-cysteinylglycine) is an important component of the plant antioxidant system that protects against the harmful effects of uncontrolled oxidation (Noctor and Foyer, 1998; Noctor et al., 2013). Moreover, GSH acts downstream of hydrogen peroxide in mediating the stress responses of phytohormones such as jasmonate and salicylate (Mhamdi et al., 2010). Many studies have implicated GSH in biotic and abiotic stress tolerance (Noctor and Foyer, 1998; Ogawa, 2005).

The pathway of GSH synthesis involves two ATP-dependent steps catalyzed by g-glutamate-cysteine ligase (GCL; also called γ-glutamylcysteine (γ-EC) synthetase), which is considered to be the rate-limiting enzyme of GSH production, and GSH synthetase (GSHS; also called GSH synthase). In *A. thaliana*, the GCL protein is found only in chloroplasts and other plastids, whereas the GSH-S is found in both the chloroplasts and cytosol (Wachter et al., 2005).

Plant homologs of the malaria chloroquine-resistance transporter *Pf*CRT (CLTs) are thiol transporters required for transport of γ-EC and GSH across the plastid envelope membranes and interconnect the plastidic and cytosolic thiol pools (Maughan et al., 2010). *Arabidopsis* mutants lacking these transporters (*clt1clt2clt3*) show enhanced sensitivity to cadmium and to the fungal pathogen, *Phytophthora brassicae*, as well as a failure to activate appropriate pathogen defense responses despite having wild-type levels of GSH in the leaves (Maughan et al., 2010). The *clt1clt2clt3 *mutants have an altered partitioning of GSH between plastid and cytosol, with a marked decrease in the cytosolic GSH levels but not in the chloroplast GSH pool in the leaves (Maughan et al., 2010). The cytosolic GSH pool is important in the mediation of systemic acquired resistance responses linked to salicylic acid signaling, as demonstrated by defects in pathogen-resistance and the expression of pathogenesis-resistance protein (PR)1 in the *clt1clt2clt3* triple mutants (Maughan et al., 2010) and in mutants that are defective in the cytosolic/peroxisomal form of NADPH-dependent glutathione reductase (*gr1*). These mutants accumulate less salicylic acid with lower *PR1* transcripts under oxidative stress conditions (Mhamdi et al., 2010). Mutants lacking the chloroplast/mitochondrial *GR2* are embryo-lethal (Tzafrir et al., 2004) but the *gr1* knockout mutants do not show a marked phenotype (Marty et al., 2009; Mhamdi et al., 2010). However, crossing the *gr1* knockout mutants with a mutant that is defective in the photorespiratory form of catalase* cat2, *led to a large accumulation in GSSG relative to the parent lines (Mhamdi et al., 2010). The *cat2 gr1 *double mutants that are deficient in both the major leaf catalase isoform and GR1 have altered responses to pathogens and expression of genes involved in jasmonate and salicylate signaling pathways (Mhamdi et al., 2010).

Glutathione synthesis and accumulation are increased in response to oxidative stress (Queval et al., 2009) because of direct effects of oxidation on the GCL protein, which is most active in its homodimeric form requiring linkage through two disulfide bonds (Hothorn et al., 2006). Reducing conditions disrupt one of the two disulfide bonds (Cys178–Cys398) altering the dimer interface and shifting the protein to the less active monomeric form (Jez et al., 2004; Hothorn et al., 2006; Galant et al., 2011). Oxidation-dependent decreases in cellular GSH/GSSG ratios also favor increased synthesis of cysteine, which is also considered to be a limiting factor for GSH synthesis (Noctor et al., 2012).

*Arabidopsis* mutants, with defects in the pathway of GSH synthesis, have been particularly useful in the characterization of GSH functions in plants. A number of mutants with defects in the *GSH1* gene that encodes GCL have been identified and these were often first characterized in terms of effects on stress tolerance. For example, the *cad2-1 *mutant that has 15–30% of wild-type GSH was identified by its enhanced sensitivity to cadmium (Cobbett et al., 1998), the *rax1-1 *mutant, where the leaf GSH pool is decreased by between 50 and 80% relative to the wild type, was identified by the altered expression of the gene encoding the cytosolic ascorbate peroxidase (Ball et al., 2004) and the *pad2-1 *mutant, where leaf GSH is decreased by 80% compared to the wild type, shows enhanced sensitivity to fungal pathogens such as *P. brassicae* and *Pseudomonas syringae* because of decreased camalexin content (Parisy et al., 2007). Mutations in the* GSH2* gene that encodes GSHS have also been very useful in elucidating the functions of glutathione in plants (Pasternak et al., 2008; Au et al., 2012). The morphology of the endoplasmic reticulum is altered and protein export is perturbed when γ-glutamylcysteine accumulates as a result of limitations in GSHS activity (Au et al., 2012). This may explain why the levels of γ-glutamylcysteine are very low in plant cells (Noctor et al., 1998).

In addition, to its antioxidant and signaling functions, GSH is also required for plant growth and development. GSH deficiency leads to an arrest in cell proliferation and root meristem formation (Vernoux et al., 2000; Diaz-Vivancos et al., 2010). GSH also has roles in flower development and vernalization responses (Bashandy et al., 2010; Hatano-Iwasaki and Ogawa, 2012). Despite this, the role of GSH in the control of plant growth under abiotic stress conditions is largely unknown.

Phenomics technologies allow accurate measurements of leaf area in large numbers of plants grown in either the absence or presence of abiotic stress over periods of days to weeks. This approach was therefore used to explore the effects of GSH deficiency on rosette leaf area in different GSH synthesis mutants (*cad2-1*, *rax1-1*, and *pad2-1*) and in the *clt1clt2clt3* triple mutants, which have altered intracellular partitioning of GSH between the chloroplasts and cytosol, under either standard (optimal) growth conditions and under abiotic stress conditions. The findings show that in contrast to the GSH synthesis mutants, which have a lower leaf area in the absence of stress, leaf area was increased in the *clt1clt2clt3* triple mutants relative to wild-type controls. The abiotic stress-induced decreases in leaf area were similar in all genotypes in short-term experiments. However, in the longer term stress treatments, the negative impacts of some abiotic stresses on leaf area were less marked in the GSH synthesis mutants than in the wild-type plants or in the *clt1clt2clt3* mutants.

## MATERIALS AND METHODS

### PLANT MATERIAL

Seeds for wild-type *A. thaliana* accession Columbia 0 (Col-0), *cad2-1* (Cobbett et al., 1998), *pad2-1* (Parisy et al., 2007), *rax1-1* (Ball et al., 2004), *rml1-1* (Cheng et al., 1995; Vernoux et al., 2000), and *clt1clt2clt3* triple mutants (Maughan et al., 2010), were sown on plates containing half strength Murashige and Skoog medium plus 1.2% glucose. Plants were grown in controlled environmental cabinets under an irradiance of 100 μmol m^-^^2^ s^-^^1^ with a photoperiod of 16 h, a constant temperature of 22 ± 2°C, and a relative humidity of 60%, for up to 17 days. For high light treatments, seedlings were grown for 10 days as above and then transferred to 400 μmol m^-^^2^ s^-^^2^ irradiance conditions for a further 4 days. The seedlings were then transferred back to 100 μmol m^-^^2^ s^-^^1^ for a further 3 days. Each experiment consisted of four plates (32 seeds per plate) per genotype and per stress treatment. Each experiment was repeated at least three times.

### SHORT STRESS TREATMENTS

For these experiments, seeds were sown on a sterile 1 μm filter mesh, which was placed on the media prior to sowing. Seedlings were grown for 10 days under the 100 μmol m^-^^2^ s^-^^1^ irradiance conditions and then transferred with the mesh to plates containing media alone (control) or growth media plus either hydrogen peroxide (4 mM), sodium chloride (75 mM), *N*,*N*’-dimethyl-4,4’-bipyridinium dichloride (paraquat; 1 μM), or sorbitol (100 mM). Seedlings were then grown for a further 7 days under these conditions and growth analysis performed as described below.

### LONG STRESS TREATMENTS

For these experiments, seeds were sown on plates in media alone (control), or media containing paraquat (0.1 μM), sodium chloride (75 mM), or sorbitol (100 mM). Seedlings were grown for 14 days under these conditions. Lower concentrations of paraquat were used in the long-term stress treatments than in the short-term stress treatments because preliminary experiments had shown that the higher concentrations of paraquat were lethal in long-term experiments. In contrast, the lower concentrations of paraquat had little effect on leaf area in the short-term experiments.

### LEAF AREA DETERMINATION

Leaf area measurements were performed as described by Schulz et al. (2012). Photographs were taken with a Canon EOS 450 D (Canon Inc., Tokyo, Japan) on successive days after sowing. Data presented here are taken from measurements made 14 and 17 days after sowing only. Total rosette surface area (hereafter called leaf area) was measured and analyzed using Fiji ImageJ^1^ as described by Schindelin et al. (2012). Data was processed using Microsoft Excel 2010 and statistical analysis was performed with program R^2^ (Hornik, 2013). The ImageJ analysis used here to calculate leaf area measures the total rosette (leaf) surface, which is highly correlated with the fresh weigh of the plant (usually *r* = 0.9–0.95).

### ROOT GROWTH AND ROOT ARCHITECTURE DETERMINATION

Primary root length and number of lateral roots were measured on 7-day-old seedlings. Root measurements were determined using Fiji ImageJ software, as above. Lateral root density was calculated from these values as the ratio between the number of visible lateral roots and the primary root length.

### METABOLITE ANALYSIS

For these experiments, the wild-type and *clt1clt2clt3* plants were grown in pots containing compost (Levington, Bramford, UK) in controlled environment chambers (16/8 h light/dark regime with photosynthetic photon flux of 250 μmol m^-^^2^ s^-^^1^). The whole rosettes of 5-week-old plants were harvested and assayed for ascorbate, glutathione, and pyridine nucleotides as described by Pellny et al. (2009).

## RESULTS

**FIGURE 1:**
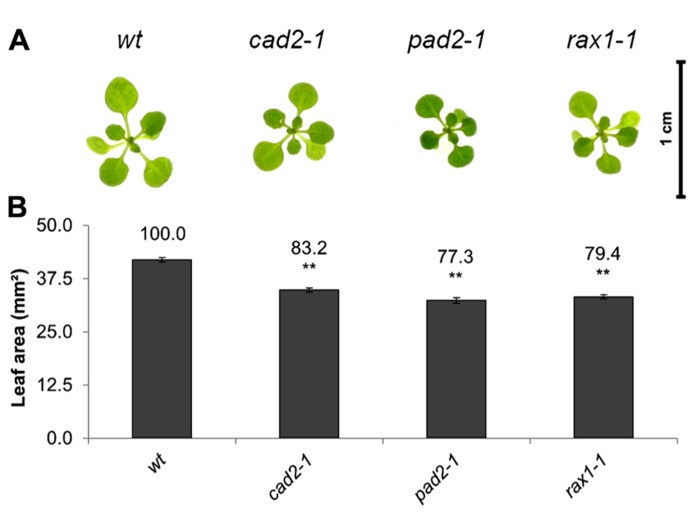
**A comparison of rosette leaf area in the *rax1-1*, cad2*-1*, and *pad2-1* mutants relative to wild type, Col-0 (wt).** Phenotypes **(A)** and leaf area of seedlings at 14 days **(B)**. The asterisks indicate significant differences (*p* < 0.05; ANOVA).

The open-source platform for biological-image analysis used in these studies involves ImageJ analysis measurements of the total rosette (leaf) surface area (from hereon called leaf area), a parameter which is highly correlated to the fresh weigh of the plant (usually *r* = 0.9–0.95). Total leaf surface area was compared in *rax1-1*, cad2*-1*, and *pad2-1* mutants with that of wild-type *A. thaliana* seedlings at 14 days (**Figure 1**). The genotypes that are deficient in GSH synthesis were visibility smaller (**Figure 1A**) and they had a significantly lower leaf area than the wild type (**Figure 1B**).

**FIGURE 2:**
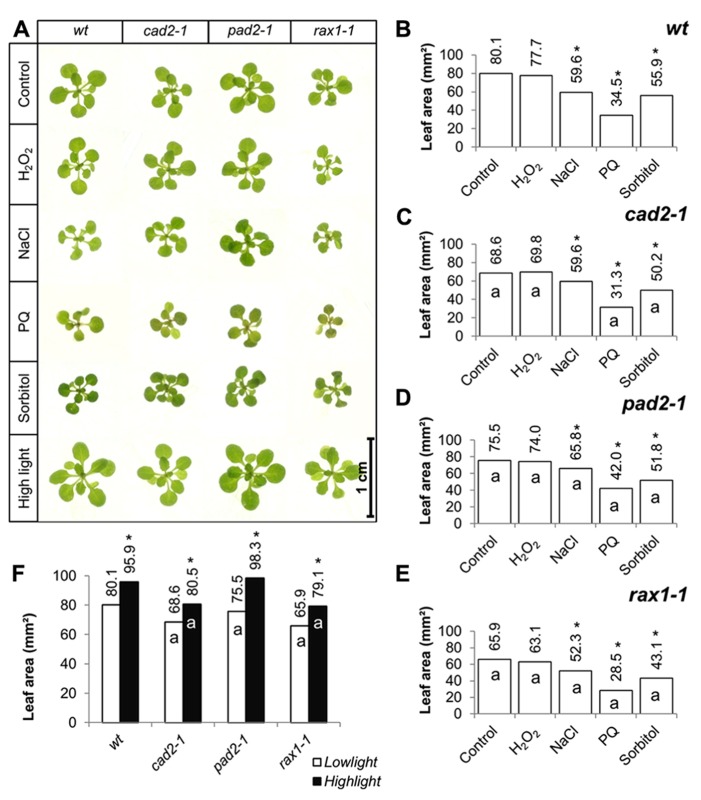
**A comparison of the effects of short-term (4 days) exposures to different abiotic stresses treatments on rosette leaf area in the *rax1-1*, cad2*-1*, and *pad2-1* mutants relative to wild type, Col-0 (wt).** In all cases seedlings were grown for 10 days under optimal conditions and then transferred to different abiotic stress treatments for a further 4 days. Phenotypes **(A)** and leaf area **(B–F)** of seedlings measured at 14 days either in the absence (control) or presence of the oxidative stress caused by the addition of hydrogen peroxide (H_2_O_2_) or paraquat (PQ), or high salt (sodium chloride, NaCl), or osmotic stress (sorbitol). For the high light treatment [closed bars, **(F)**] seedlings were either grown under 150 μmol m^-^^2^ s^-^^1^ irradiance for 14 days (open columns) or they were grown under 150 μmol m^-^^2^ s^-^^1^ irradiance for 10 days and then transferred to 400 μmol m^-^^2^ s^-^^1^ irradiance for 4 days prior to measurement. The asterisks indicate significant differences (*p* < 0.05; ANOVA).

The effects of short-term (7 days) exposure to different abiotic stress treatments on leaf area was measured (**Figure 2**). Exposure to oxidative stress (4 mM hydrogen peroxide or 1 μM paraquat), high salt (75 mM sodium chloride), or osmotic stress (100 mM sorbitol) led to a visible shoot phenotype (**Figure 2A**) and a decrease in leaf area in all genotypes (**Figures 2B–E**). All treatments except exposure to hydrogen peroxide led to a significant decrease in leaf area in all genotypes (**Figures 2B–E**). The genotypes that were defective in GSH synthesis were not more sensitive to the treatments in relation to leaf area than the wild type except for the *rax1-1*, which was more sensitive to the paraquat treatment (**Figure 2E**). However, the *pad2-1* mutants were less sensitive to the paraquat treatment relative to the wild type (**Figure 2D**). The *cad2-1* and *pad2-1* mutants were also less sensitive to the high salt treatment than the wild type (**Figure 2C**). Short-term (4 days) exposure to a relatively high light (400 μmol m^-^^2^ s^-^^1^) treatment visibly stimulated increases in leaf area (**Figure 2A**) and led to a significant increase in leaf area in all genotypes (**Figure 2F**). Moreover, under these conditions the *pad2-1* mutants had a similar leaf area to the wild type, whereas the leaf area in the *cad2-1* and *rax1-1 *plants was significantly smaller than the wild type (**Figure 2F**).

**FIGURE 3:**
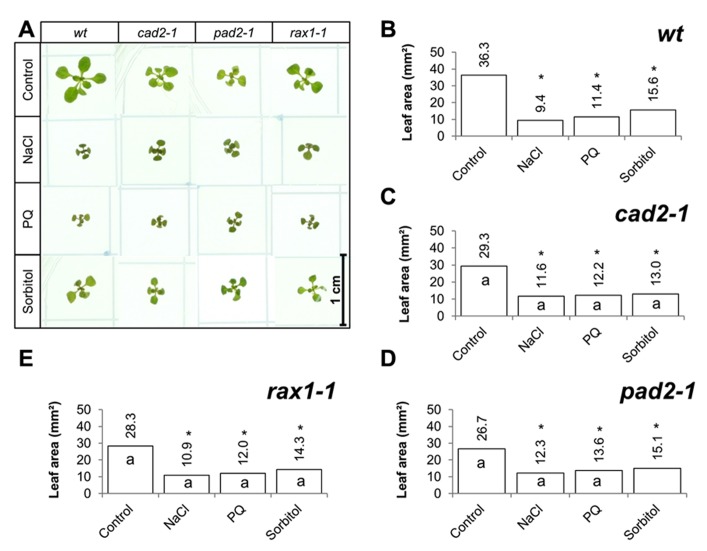
**A comparison of the effects of long-term (14 days) exposures to different abiotic stresses treatments on rosette leaf area in the *rax1-1*, cad2*-1*, and *pad2-1* mutants relative to wild type, Col-0 (wt).** Phenotypes **(A)** and leaf area **(B–E)** of seedlings measured at 14 days either in the absence (control) or presence of the oxidative stress caused by the addition of paraquat (PQ) or high salt (sodium chloride, NaCl), or osmotic stress (sorbitol). The asterisks indicate significant differences (*p* < 0.05; ANOVA).

The effects of long-term (14 days) exposure to the different abiotic stress treatments such as oxidative stress (paraquat), salt stress (sodium chloride), or osmotic stress (sorbitol) on leaf area was measured in all genotypes (**Figure 3**). All treatments led to a visible decrease in the rosettes of all genotypes (**Figure 3A**) and significant decrease in leaf area in all cases (**Figures 3B–E**). In contrast to the osmotic stress treatment, which led to similar decrease in leaf area in all genotypes except the *pad2-1* mutants, the GSH synthesis mutants had a significantly greater leaf area than the wild type in the oxidative stress treatment (paraquat) and in the high salt treatment (**Figures 3B–E**).

**FIGURE 4:**
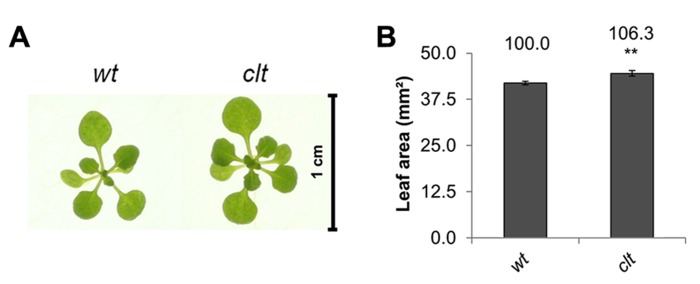
**A comparison of rosette leaf area in the *clt1clt2clt3* triple mutants (*clt*) relative to wild type, Col-0 (wt).** Phenotypes **(A)** and leaf area of seedlings at 14 days (**B**; **p < 0.01).

**FIGURE 5:**
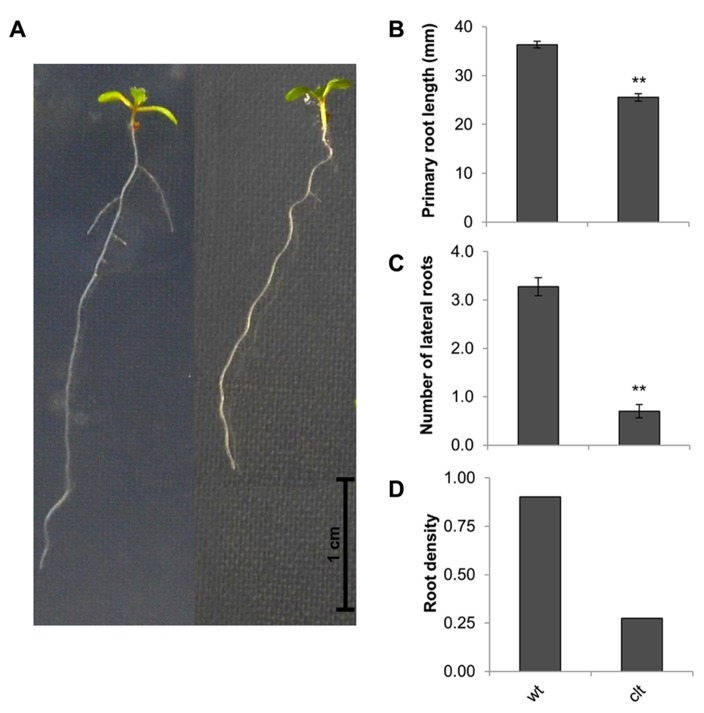
**Root architecture the *clt1clt2clt3* triple mutants (*clt*) relative to wild type Col-0 (wt).** The phenotype of 14-day-old seedlings **(A)**, primary root length **(B)**, number of visible lateral roots **(C)**, and lateral root density **(D)**. The asterisks indicate significant differences (*p* < 0.05; ANOVA).

The ascorbate and glutathione contents of the *clt1clt2clt3* leaves were similar to that of the wild type (**Table 1**). The rosettes of the *clt1clt2clt3* triple mutants were visibly larger than those of the wild type (**Figure 4A**) and they had significantly greater leaf area (**Figure 4B**). However, root architecture was markedly different in the *clt1clt2clt3* triple mutants relative to the wild type (**Figure 5A**). The primary root length was significantly shorter than that of the wild type (**Figure 5B**) and there were significantly fewer lateral roots (**Figure 5C**). The lateral root density was markedly decreased in the *clt1clt2clt3* triple mutants relative to the wild type (**Figure 5D**).

**Table 1:** A comparison of the major redox metabolites in the rosette leaves of the *clt1clt2clt3* triple mutants and wild-type (Col-0) plants.

Metabolite	Genotype	
	Col-0	*clt1clt2clt3*
Ascorbate (μmol mg^-^^1^ Chl)	3.48 ± 0.17^a^	4.20 ± 0.18^a^
Dehydroascorbate (μmol mg^-^^1^ Chl)	1.34 ± 0.14^a^	1.01 ± 0.16^a^
Ascorbate/dehydroascorbate	2.59^a^	3.82^a^
GSH (μmol mg^-^^1^ Chl)	228.33 ± 30.10^a^	264.67 ± 30.64^a^
GSSG (μmol mg^-^^1^ Chl)	7.51 ± 0.46^a^	10.73 ± 0.81^a^
GSH/GSSG	31.23^a^	20.10^a^
NADH (μmol mg^-^^1^ Chl)	2.73 ± 0.48^a^	2.44 ± 0.16^a^
NAD (μmol mg^-^^1^ Chl)	7.12 ± 1.12^a^	5.61 ± 0.77^a^
NADH/NAD	0.38^a^	0.43^a^
NADPH (μmol mg^-^^1^ Chl)	25.59 ± 5.42^a^	16.45 ± 2.50^b^
NADP (μmol mg^-^^1^ Chl)	1.77.88 ± 0.09^a^	4.22 ± 0.40^b^
NADPH/NADP	15.02^a^	3.90^b^

*Values represent the mean values ± SE (*n* = 6). Values within a single row that were significantly different as estimated by Fishers protected LSD test (*p* < 0.05) are indicated by different letters.*

**FIGURE 6:**
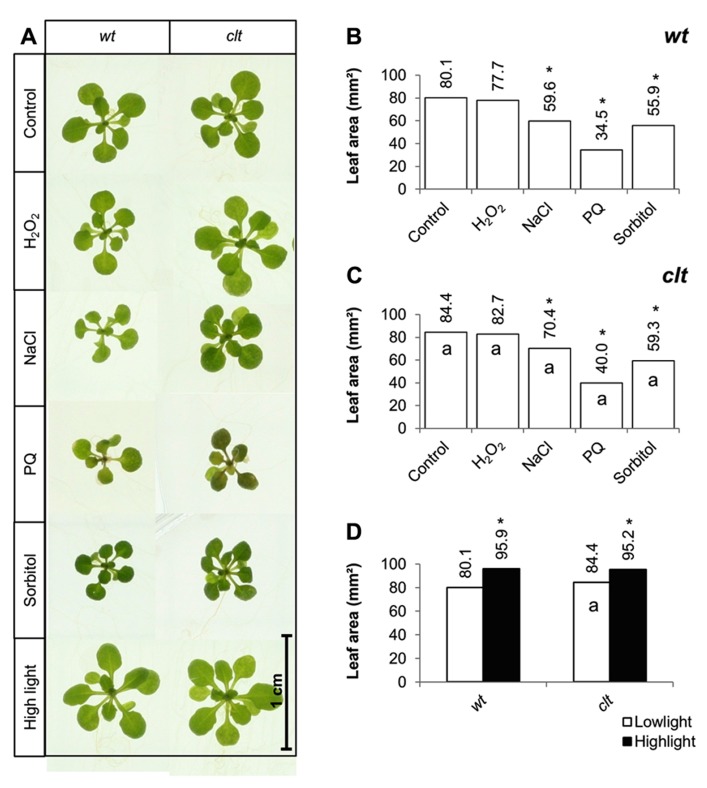
**A comparison of the effects of short-term (4 days) exposures to different abiotic stresses treatments on rosette leaf area in the *clt1clt2clt3* triple mutants relative to wild type, Col-0 (wt).** In (**A–C)** seedlings were grown for 10 days under optimal conditions and then transferred different abiotic stress treatments for a further 4 days. Phenotypes **(A)** and leaf area **(B,C)** of seedlings measured at 14 days either in the absence (control) or presence of the oxidative stress caused by the addition of hydrogen peroxide (H_2_O_2_) or paraquat (PQ), or high salt (sodium chloride, NaCl), or osmotic stress (sorbitol). For the high light treatment (closed bars, **(D)**) seedlings were either grown under 150 μmol m^-^^2^ s^-^^1^ irradiance for 14 days (open columns) or they were grown under 150 μmol m^-^^2^ s^-^^1^ irradiance for 10 days and then transferred to 400 μmol m^-^^2^ s^-^^1^ irradiance for 4 days prior to measurement. The asterisks indicate significant differences (*p* < 0.05; ANOVA).

The effects of short-term (7 days) exposure to abiotic stress on leaf area were compared in the *clt1clt2clt3* triple mutants and wild-type plants (**Figure 6A**). Exposure to low levels of hydrogen peroxide (4 mM) had no effect on leaf area in either genotype (**Figure 6**). Salt stress caused a significant decrease in leaf area in both genotypes, but the salt-induced decrease in leaf area was greater in the wild type than the *clt1clt2clt3* triple mutants (**Figures 6B,C**). Similarly, exposure to paraquat caused a significant decrease in leaf area in both genotypes, but the oxidative stress-induced decrease in leaf area was greater in the wild type than the *clt1clt2clt3* triple mutants (**Figures 6B,C**). Moreover, exposure to osmotic stress caused a significant decrease in leaf area in both genotypes, but the osmotic stress-induced decrease in leaf area was greater in the wild type than the *clt1clt2clt3* triple mutants (**Figures 6B,C**). Short-term (4 days) exposure to a high light resulted in visibly larger rosettes (**Figure 6A**) and led to a significant increase in leaf area in all genotypes (**Figure 6D**).

**FIGURE 7:**
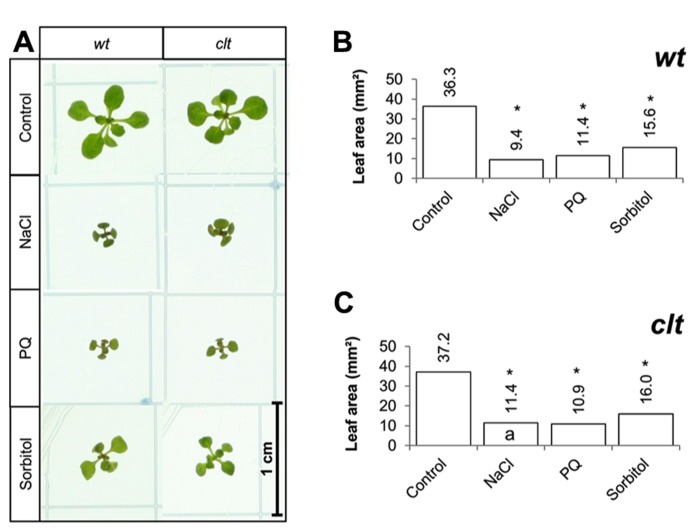
**A comparison of the effects of long-term (14 days) exposures to different abiotic stresses treatments on rosette leaf area in the *clt1clt2clt3* triple mutants relative to wild type, Col-0 (wt).** Phenotypes **(A)** and leaf area **(B,C)** of seedlings measured at 14 days either in the absence (control) or presence of the oxidative stress caused by the addition of paraquat (PQ) or, or high salt (sodium chloride, NaCl), or osmotic stress (sorbitol). The asterisks indicate significant differences (*p* < 0.05; ANOVA).

The effects of long-term (14 days) exposure to the different abiotic stress treatments were compared in the *clt1clt2clt3* triple mutants and wild-type plants (**Figure 7A**). All treatments led to a visible decrease in the size of the rosettes of both genotypes (**Figure 7A**). A significant decrease in leaf area was observed following exposure to oxidative stress, high salt, and osmotic stress in both genotypes (**Figures 7B,C**).

The total leaf ascorbate or glutathione contents were similar in the *clt1clt2clt3* triple mutants and the wild type (**Table 1**). While the NAD^+^/NADH ratios were also similar in the leaves of both genotypes, the *clt1clt2clt3* triple mutant leaves had higher NADP^+^/NADPH ratios than those of the wild type (**Table 1**).

## DISCUSSION

Genetic evidence has demonstrated links between glutathione redox state and shoot and root meristem activity (Vernoux et al., 2000; Reichheld et al., 2007; Bashandy et al., 2010; Koprivova et al., 2010). However, mechanisms by which GSH participate in the control of growth particularly under stress conditions remain to be characterized. The *rml1-1* mutant, which has less than 5% of the wild-type GSH levels is unable to establish a post-embryonic root meristem because of cell cycle arrest at G1 (Vernoux et al., 2000). Similarly, a pharmacological approach has demonstrated that inhibition of GSH synthesis also leads to an arrest of root growth (Koprivova et al., 2010). Under similar growth conditions to those used here the *cad2-1*, *pad2-1*, and *rax1-1* mutants had a lower number of lateral roots leading to a lower lateral root density in all the GSH deficient mutant genotypes compared to the wild type (Marquez-Garcia et al., 2013). While visual inspection failed to establish a clear phenotype under routine growth conditions (Maughan et al., 2010), the results presented here clearly demonstrate that like the GSH synthesis mutants, the *clt1clt2clt3* triple mutants have significantly lower lateral root densities than the wild type. These data indicate that a high cytosolic GSH pool is required in the control of root architecture. Root growth may be regulated in part by the shoot; the decrease in the cytosolic GSH levels in *clt1clt2clt3* leaves leading to GSH depletion in the roots, which rely, at least in part, on GSH transport from the leaves (Noctor et al., 1998; Li et al., 2006). Moreover, whereas the *cad2-1*, *rax1-1*, and the *pad2-1 *mutants have decreased leaf area relative to the wild type, leaf area shows a small but significant increase in the *clt1clt2clt3* triple mutants compared to the wild type. These data may implicate chloroplast GSH pool in the regulation of leaf area. However, rosette size was significantly enhanced by high light, which led to a significant increase in leaf area in all genotypes. Under these conditions the *pad2-1* mutants performed better than the *cad2-1* and *rax1-1 *mutants, achieving leaf areas that were similar to or even slightly higher than the wild type, suggesting that high light can overcome the adverse influence of low GSH in signaling that controls leaf area.

The results presented here show that the leaves of the *clt1clt2clt3 *mutants had a similar redox status to the wild-type plants, under optimal growth conditions, with comparable ascorbate and glutathione levels and similar ascorbate/dehydroascorbate, GSH/GSSG, and NAD/NADH ratios, even though the partitioning of GSH between the cytosol and chloroplasts was changed in the *clt1clt2clt3 *mutants relative to the wild type (Maughan et al., 2010). The observed decreases in the leaf NADPH/NADP^+^ ratios in the *clt1clt2clt3* mutants linked to the altered intracellular partitioning of GSH between these compartments, might be explained by in terms of increased demand for NADPH for cytosolic redox processes linked to thioredoxin, as a result of GSH depletion (Marty et al., 2009).

Short-term exposures to oxidative stress (paraquat), salt stress, and osmotic stress resulted in a decrease in leaf area in all genotypes. However, the stress effects were similar in the GSH synthesis mutants and in the *clt1clt2clt3* triple mutants to the wild type, the only exception being the *rax1-1 *mutant, which was slightly more sensitive to the paraquat treatment. Longer term abiotic stress treatments caused larger decreases in leaf area in all genotypes. However, in contrast to the *clt1clt2clt3* triple mutants, which showed a similar response to the abiotic stresses to the wild-type plants, the GSH synthesis mutants with the exception of *pad2-1*, had a significantly greater leaf area than the wild type under the oxidative stress and the high salt treatments. These results suggest that impaired GSH synthesis capacity may therefore serve to mitigate the adverse effects of some abiotic stresses such as salt stress and dehydration on leaf growth but not others such as heavy metal stress, where GSH is required for other pathways such as phytochelatin biosynthesis.

The observation that GSH deficiency limits the adverse effects of salt stress and dehydration on leaf growth may be related to the central role of glutathione in the regulation of gene expression linked to oxidative stress signaling (Noctor et al., 2013). Accumulating evidence supports the concept that glutathione status is involved in the cross talk between oxidative signaling and hormone signaling (Mhamdi et al., 2010; Han et al., 2013a,b). Crucially, glutathione status also influences the auxin signaling pathways that control growth (Bashandy et al., 2010; Gao et al., 2013). The oxidative signals that limit growth under oxidative stress are therefore likely to be transmitted at least in part via modulation of the redox status of the glutathione pool. The greater leaf area observed here in the GSH synthesis mutants relative to the wild type under oxidative and high salt stresses may therefore be linked to a requirement for GSH in the cross talk between redox and hormone-mediated signaling processes that serve to restrict growth in plants exposed to abiotic stress.

## CONCLUSION

Abiotic stress tolerance is an important factor determining plant growth and productivity, and is the subject of ever-intensifying interest in relation to crop improvement. The importance of antioxidants such as glutathione in abiotic stress tolerance is well documented, particularly with regard to its antioxidant functions in protection against stress-induced oxidation. In addition to its potential usefulness as a stress marker, glutathione status is important in the control of growth and oxidative stress signaling (Noctor et al., 2013). Within this context, the results presented here demonstrates that the intracellular compartmentalization of glutathione influences plant growth, a depletion in the cytosol in the *clt1clt2clt3* triple mutants leading to significant decreases in lateral root density and increases in rosette leaf area under non-stressed conditions. However, in contrast to biotic stress tolerance, which is impaired in the *clt1clt2clt3* triple mutants (Maughan et al., 2010), depletion of the cytosolic GSH pool had no effect on the stress-induced decreases in leaf area in plants experiencing short or long periods of abiotic stress. Conversely, while decreases in GSH synthesis capacity resulted in significant decreases in lateral root density (Marquez-Garcia et al., 2013), this change in root architecture was accompanied by decreased rosette leaf area under non-stressed conditions. Moreover, limitations on GSH synthesis capacity favored larger leaf areas in plants experiencing long (but not short) periods of abiotic stress. Taken together, these findings shed new light on the functions of glutathione in plant growth and abiotic stress tolerance, showing that unexpectedly limitations on GSH synthesis enhance abiotic stress tolerance in the longer term as determined by leaf area. Moreover, while the intracellular partitioning of glutathione is important in the regulation of root architecture, it has little impact on leaf area and hence abiotic stress tolerance.

## Conflict of Interest Statement

The authors declare that the research was conducted in the absence of any commercial or financial relationships that could be construed as a potential conflict of interest.

## Footnotes

1 http://fiji.sc/

2 www.r-project.org
